# Blocking NMDA-receptors in the pigeon’s “prefrontal” caudal nidopallium impairs appetitive extinction learning in a sign-tracking paradigm

**DOI:** 10.3389/fnbeh.2015.00085

**Published:** 2015-04-13

**Authors:** Daniel Lengersdorf, David Marks, Metin Uengoer, Maik C. Stüttgen, Onur Güntürkün

**Affiliations:** ^1^Faculty of Psychology, Department of Biopsychology, Institute of Cognitive Neuroscience, Ruhr University BochumBochum, Germany; ^2^Department of Psychology, Philipps-University MarburgMarburg, Germany; ^3^Institute of Pathophysiology, University Medical Center of the Johannes Gutenberg UniversityMainz, Germany; ^4^Focus Program Translational Neuroscience, University Medical Center of the Johannes Gutenberg UniversityMainz, Germany

**Keywords:** renewal, APV, sign-tracking, context, retrieval

## Abstract

Extinction learning provides the ability to flexibly adapt to new contingencies by learning to inhibit previously acquired associations in a context-dependent manner. The neural networks underlying extinction learning were mostly studied in rodents using fear extinction paradigms. To uncover invariant properties of the neural basis of extinction learning, we employ pigeons as a model system. Since the prefrontal cortex (PFC) of mammals is a key structure for extinction learning, we assessed the role of N-methyl-D-aspartate receptors (NMDARs) in the nidopallium caudolaterale (NCL), the avian functional equivalent of mammalian PFC. Since NMDARs in PFC have been shown to be relevant for extinction learning, we locally antagonized NMDARs through 2-Amino-5-phosphonovalerianacid (APV) during extinction learning in a within-subject sign-tracking ABA-renewal paradigm. APV-injection slowed down extinction learning and in addition also caused a disinhibition of responding to a continuously reinforced control stimulus. In subsequent retrieval sessions, spontaneous recovery was increased while ABA renewal was unaffected. The effect of APV resembles that observed in studies of fear extinction with rodents, suggesting common neural substrates of extinction under both appetitive and aversive conditions and highlighting the similarity of mammalian PFC and the avian caudal nidopallium despite 300 million years of independent evolution.

## Introduction

Learning enables organisms to survive in a permanently changing environment. During learning, stimuli that are initially neutral become associated with co-occurring unconditioned stimuli and acquire the ability to elicit conditioned responses. Extinction learning of these conditioned responses is as relevant for adaptive behavior as initial acquisition. During extinction, a conditioned stimulus appears repeatedly without the unconditioned stimulus, and subsequently the conditioned response vanishes. Unlike original acquisition, extinction learning is highly context-dependent. After successful extinction, the transfer to a context other than that where extinction took place results in the reappearance of the conditioned behavior, a phenomenon termed renewal (Bouton and Bolles, 1979; Bouton and Ricker, 1994; Rauhut et al., 2001; Bouton, 2002; Crombag and Shaham, 2002). It illustrates that extinction does not simply erase the old memory trace but entails new learning (Pavlov, 1927; Bouton, 2004). The majority of studies on extinction learning employ fear conditioning experiments in rodents (Quirk and Mueller, 2008; Quirk et al., 2010). Results from both rodent and human studies point to three prominent brain areas as critical for extinction learning: amygdala, prefrontal cortex (PFC) and hippocampus. Contextual information is integrated by the hippocampus while the PFC and its interactions with substructures of the amygdala seem to play a key role in extinction organization and retrieval (Hobin et al., 2003; Peters et al., 2009). In rats, the functionality of the PFC for extinction learning differs between two main subareas, prelimbic and infralimbic PFC, which seem to have opposite functions. While the infralimbic cortex facilitates extinction learning, prelimbic cortex seems to inhibit it (Milad and Quirk, 2012). Pharmacological manipulations of the hippocampus as well as the PFC demonstrate that these structures are involved in contextual coding during renewal and extinction retrieval (Corcoran and Maren, 2004; Burgos-Robles et al., 2007).

Extinction learning is an evolutionary conserved phenomenon that can be studied in vertebrates and invertebrates (Stollhoff et al., 2005). But are the neural mechanisms involved in extinction learning in other species comparable to what we know from mammals? To answer this question, we study pigeons, a species that represents a classic model organism for conditioning tasks (Skinner, 1948; Güntürkün et al., 2014) but is separated from mammals by 300 million years of evolution. The pigeon brain is devoid of a cerebral cortex, but their pallium is partly homologous to mammalian cortex. In addition, there is strong evidence that birds have a specialized pallial area, the nidopallium caudolaterale (NCL) which constitutes a functional equivalent to the mammalian PFC (reviewed in Güntürkün, 2005; Lengersdorf et al., 2014a). Recently, Lengersdorf et al. (2014b) showed that transient NCL inactivation impairs context-specific extinction memory consolidation. It is possible that the consolidation of extinction memory in the NCL is mediated via N-methyl-D-aspartate receptors (NMDARs). Indeed, Herold et al. (2011) revealed a high density of NMDAR in the pigeon’s NCL, and Lissek and Güntürkün (2003) observed that the injection of 2-Amino-5-phosphonovalerianacid (APV), a NMDAR antagonist, in the NCL resulted in impaired extinction learning. Moreover, Lissek and Güntürkün (2005) provided evidence for the role of NCL NMDARs in contextual processing in a conditional discrimination task. In those studies, however, the possible contextual dependency of NMDARs in the NCL for extinction learning was not assessed. Therefore, we adapted this treatment to our established within-subject context-dependent extinction task for pigeons (Lengersdorf et al., 2014b). Bilateral injection of the NMDR antagonist APV in the NCL before extinction training was thus employed to test the hypothesis that the blockade of NMDAR in the NCL impairs extinction learning.

## Materials and Methods

### Subjects

Adult unsexed pigeons (*Columba livia*) served in both experiments. Overall twenty-one animals participated in the experiment. Subjects were housed singly in wire-mesh cages (30 cm × 30 cm × 45 cm) in a colony room, with a 12-h light-dark schedule (lights on 8 a.m.), controlled humidity and temperature. The access to water was ad libitum while access to food was restricted (see below). Body weight was monitored daily and maintained around 85% of the free-feeding weight. All experiments were approved by the national authorities of the state of North Rhine-Westphalia, Germany and carried out in accordance with the National Institute of Health Guide for Care for Laboratory Animals.

### Surgery

Naïve pigeons were prepared for bilateral cannula implantation with the painkiller Dolorex (0.3 ml, 10 mg/ml, Butorphanol, Intervet, MSD Animal Health, Unterschleißheim, Germany). Gas anesthesia (Isoflorane; Forane 100% (V/V), Mark 5, Medical Developments International, Abbott GmbH and Co KG, Wiesbaden, Germany) was initiated 10–15 min after painkiller injection. Feathers on top of the skull were cut, the skin was removed, and 8–10 stainless steel microscrews (Small Parts, Logansports, USA) were placed on the skull to anchor head mounts. Additionally, two small craniotomies were performed above the target areas to provide access to the underlying brain tissue. One double cannula (26-gauge, length 8 mm, spaced 2 mm, Plastics One Inc., Roanoke, USA) was inserted into each hemisphere under visual control at the following coordinates: AP +5.25 mm, L ±5 and 7 mm, V +1.1 mm (Karten and Hodos, 1967) at an angle of 30° relative to the coronal plate. Dental cement was used to fixate the cannulas at the defined position. Following surgery, injections of the painkiller Carprofen (0.3 ml, 10 mg/ml, Rimaldyl, Pfizer GmbH, Münster, Germany) were administered twice daily for at least 3 days. Animals were allowed to recover for 7–10 days following surgery before initial training commenced.

### Behavioral Apparatus

Training was conducted in four similarly shaped experimental chambers (36 cm × 34 cm × 36 cm). Each chamber was placed in a sound-attenuating cubicle. White or brown noise (approximately 80 dB SPL) was played continuously to mask extraneous sounds. The center of the rear wall consisted of a transparent plexiglass pecking key (2 cm × 2 cm; 12 cm above the floor) to measure key pecking responses. Each registered response produced an audible feedback click. Stimuli were presented on LCD flat screen monitors mounted behind the chambers (2 × Belinea Model No.: 101536; Philips Model No. 150S4 and Model No. 150P4CG/00), hence a stimulus on the monitor was visible through the plexiglass pecking key. A food hopper was positioned at the bottom center underneath the pecking key. The internal illumination of the boxes was provided either by 6W light bulbs or LED bands at the ceiling. Distinct contexts were produced by covering the rear and the side walls of the chambers with different color cards: Either by 2.5 cm wide vertical tan stripes spaced 5 cm apart on red background, or by yellow marbling pattern on white background. Four stimuli with different color patterns were used in each experiment. The hardware was controlled by custom-written Matlab code (The Mathworks, Natick, MA; Rose et al., 2008).

### Procedure

**Figure 1:**
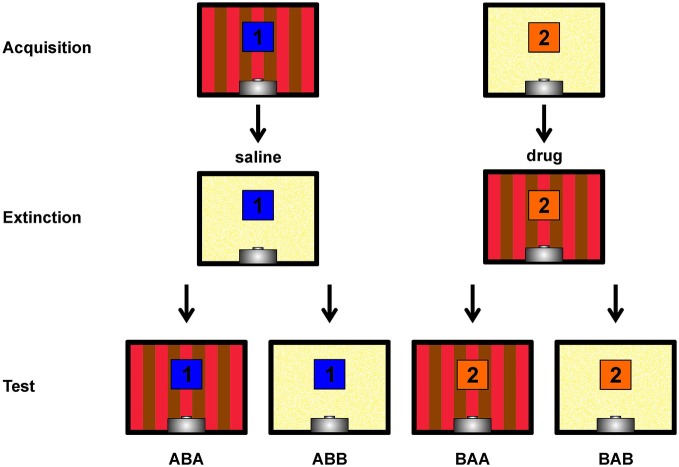
**Depiction of the within-subject ABA renewal design**. Single pictures show rear walls of the two different conditioning chambers **(A,B)**. The blue and orange squares with numbers 1 and 2 indicate the two different conditioned stimuli. Not shown are the target stimulus (present and reinforced in all sessions) and the non-target stimulus (present and non-reinforced in all sessions). Contexts, stimuli and injection sequences were balanced across subjects, hence this figure shows a single possible example. Figure bases on Lengersdorf et al. (2014b).

The complete experiment included five different phases labeled Pretraining I, Pretraining II, Acquisition, Extinction and Test. Details of each experimental stage will be explained below and are illustrated in Figure 1, Table 1.

**Table 1:** **General training procedure overview. ((+) = rewarded stimulus; (−) = non-rewarded stimulus; CS1 = conditioned stimulus 1; CS2 = conditioned stimulus 2; — = no stimulus presentation)**.

Phase	Context	No. target	No. non-target	No. CS1 or CS2
**Pretraining I**	A	48x (+)	—	—
	B	48x (+)	—	—
**Pretraining II**	A	24x (+)	12x (−)	—
	B	24x (+)	12x (−)	—
**Acquisition**	A	12x (+)	12x (−)	12x CS1 (+)
	B	12x (+)	12x (−)	12x CS2 (+)
**Extinction**	A	24x (+)	12x (−)	24x CS2 (−)
	B	24x (+)	12x (−)	24x CS1 (−)
**Test**	A	12x (+)	12x (−)	12x CS1 (−) and 12x CS2 (−)
	B	12x (+)	12x (−)	12x CS1 (−) and 12x CS2 (−)

#### Pretraining I

Animals were trained on a simple sign tracking task (a Pavlovian conditioning procedure sometimes also referred to as autoshaping; Brown and Jenkins, 1968). A stimulus (“target”) appeared for 5 s. Upon termination of the stimulus, the food hopper was activated to provide grain for 3 s. The trials were separated by a fixed intertrial interval of 45 s. Responses during stimulus presentation were counted. Each session contained 48 target presentations. Training was conducted twice daily (work days only), once in each context. Sessions were spaced 2 h apart, and the context sequence (A→B or B→A) alternated daily. Once an animal exhibited conditioned responding in at least 80% of the trials in both contexts, the subject entered the next training stage (Pretraining II).

#### Pretraining II

The conditions of Pretraining I were extended by introducing 12 presentations of a non-reinforced stimulus (“non-target”). The number of target presentations was reduced to 24, and the duration of the intertrial interval was reduced to 35 s. Each session started with two target presentations, followed by randomized stimulus presentation. Conditioned responding in at least 80% of target and non-responding in at least 80% of non-target trials was required for the animal to move into the next training phase (Acquisition).

The two stimuli employed in the two separate Pretraining phases served to detect possible non-systematic effects (up- or downregulations of responding) brought upon by pharmacological treatments during extinction. Additionally, the non-target served to discourage pigeons from responding indiscriminately to the visual stimuli. To summarize, the target stimulus was always followed by reward while the non-target was never followed by reward, and these contingencies were maintained throughout the entire experiment.

#### Acquisition

In this phase, three different stimuli (target, non-target, and CS1 or CS2, depending on the context) were presented in random order, each for 12 times. A rewarded CS1 was added in context A and a rewarded CS2 was added in context B. The performance criterion for completion of the acquisition phase was extended to a minimum of 6 days of training and three consecutive days of 80% correctly responded trials.

#### Extinction

Two extinction sessions in which either CS1 or CS2 was not followed by reinforcement anymore were conducted on separate days, spaced 48 h apart: One session with drug infusion and one with saline infusion (sequence counterbalanced). One day off between extinction days was necessary to guarantee complete washout of the drug. To adjust the daily amount of food, subjects were provided with 10 g of grain on days without training. Approximately 10–15 min before extinction commenced, either APV (total volume 2 µl, containing 10 µg of APV; 0.5 µl per cannula, i.e., 2.5 µg of APV per cannula) or saline (total volume 2 µl; 0.5 µl per cannula) was microinjected bilaterally (see Helduser and Güntürkün, 2012 for more procedural details). Irrespective of treatment, each extinction session consisted of 24 non-reinforced CS presentations, as well as 12 non-target and 24 target presentations. During extinction, CS presentation was never followed by grain and was tested in the context in which it had not been presented during acquisition training: thus, CS1 was presented in context B and CS2 was presented in context A. Since this constitutes a within-subject experimental design, all animals experienced extinction of one CS under saline and extinction of the other CS under drug conditions.

#### Retrieval Test

48 h after the second extinction session, all stimuli were presented 12 times each (randomized sequence) under drug-free conditions on a single day. Testing took place in both contexts with test sessions separated by 2 h. Each test session contained all four stimulus types (target, non-target, CS1 and CS2) and started with two target presentations. CS presentations remained unrewarded, as during extinction training. Since both CSs were presented in both contexts, ABA renewal as well as spontaneous recovery (ABB) of responding could be assessed. The character sequences ABA and ABB refer to the order of contexts in which Acquisition, Extinction and Retrieval were assessed, respectively (Figure 1).

### Histology

After completion of the test session, injection sites were verified with immunohistochemical techniques. Animals received a lethal injection of Equithesin (0.5 µl per 100 g body weight). Once the animal was deeply anesthetized and claw reflexes were completely absent, transcardial perfusion with warm sodium chloride solution (0.9%, 38°C) and subsequently cold paraformaldehyde (4% in 0.12 M phosphate buffer pH 7.4, PBS, 4°C) was performed. The brain was removed and postfixed in 4% paraformaldehyde for 2 h. Then the brain was transferred to paraformaldehyde with additional 30% sucrose overnight for cryoprotection and subsequently sliced in 40 µm sections. Sections were stained with cresyl violet to reveal anatomical structures. The position of the cannulas were analyzed under the microscope by means of the brain atlas from Karten and Hodos (Karten and Hodos, 1967).

### Data Analysis

The main dependent variable was the fraction of trials in which animals showed conditioned responding during the 5 s CS presentation interval (henceforth, “fractional response count”). This variable was chosen because results from our previous study suggested that this variable is more sensitive for detecting drug effects than the absolute number of conditioned responses. Nonetheless, absolute response counts during CS presentation were also analyzed. Statistical analyses were conducted employing one-way and two-way repeated-measures analyses of variance (RMANOVA), along with paired-samples *t*-tests. All analyses were performed with the Statistics Toolbox of Matlab R2012a (The Mathworks, Natick, USA). Normalized response counts during extinction were calculated by multiplying the average number of responses in a given bin of four consecutive trials by the ratio of target responses under saline and drug in the same bin of four trials, separately for each animal. Since animals almost never responded during presentation of the non-target stimulus, response data for this stimulus are not shown in the result figures.

## Results

### Histology

**Figure 2:**
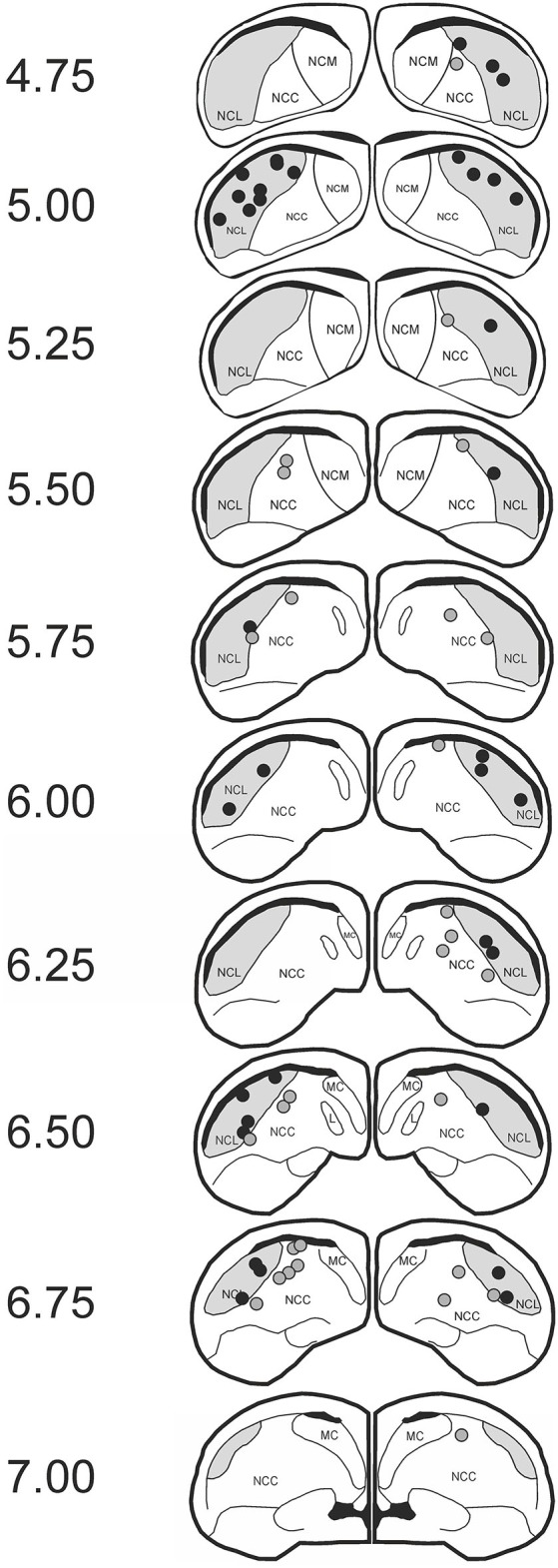
**Histological data**. Schematic slices of the pigeon brain highlighting APV injection sites. Dots represent the tips of the injection cannulas (black: nidopallium caudolaterale (NCL); gray: NCC). Pictures are based on the brain atlas by Karten and Hodos (1967).

We tested 21 subjects. Two animals were excluded due to improper cannula position, two animals failed to achieve criterion performance, and another animal was subjected to an incorrect extinction procedure due to a mistake of the experimenter, leaving 16 subjects for analysis. Regarding cannula position, subjects were included if the tip of the lateral cannulas was positioned in the NCL and the medial cannula was either in the NCL or the nidopallium caudocentrale (NCC). Overall 36 cannulas were found to be within the NCL and 28 cannulas were placed in the NCC (Figure 2). The NCC is adjacent to the NCL. As judged from the fiber connections (Rehkämper and Zilles, 1991; Husband and Shimizu, 1999; Atoji and Wild, 2009) and a lesion study (Hartmann and Güntürkün, 1998) the NCC is sketched as a tertiary limbic area. Herold et al. (2011) reported that the NMDAR density within the NCC is comparable to that of the NCL. The reported effects therefore result from manipulations of both areas.

### Acquisition

**Figure 3:**
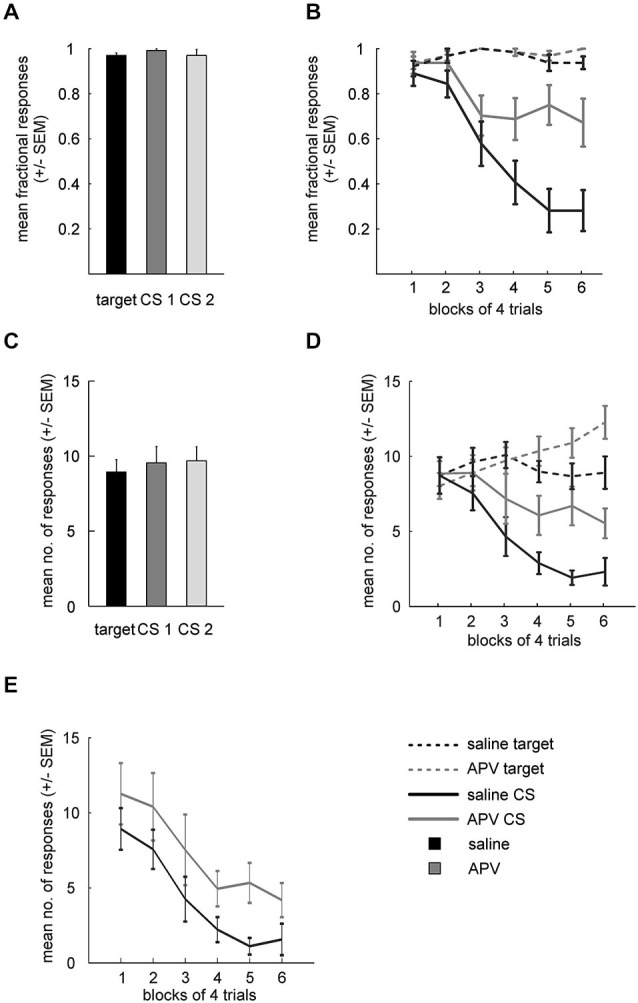
**Results from APV injections. (A)** Mean fractional response counts (±SEM) for the target and the two CS in the last three acquisition sessions. **(B)** Mean fractional response counts (±SEM) during extinction learning. Dashed and solid lines depicted data from target and CS trials, respectively. Gray lines, extinction under APV, black lines, extinction under saline. **(C)** Mean absolute response rate (±SEM) during the last 3 days of acquisition. **(D)** Absolute response counts mirror results from fractional response counts and additionally indicate unspecific disinhibition of conditioned responding. **(E)** Normalized response counts reveal prolonged extinction for APV treated subjects.

Mean fractional response rates for individual stimuli during acquisition over the last three sessions were similar (Figure 3A) and accordingly did not differ significantly (paired *t*-test: target vs. CS1: *t*_(15)_ = 1.7; *p* = 0.111; target vs. CS2: *t*_(15)_ = 0.4; *p* = 0.693; CS1 vs. CS2: *t*_(15)_ = 1.14; *p* = 0.27).

Absolute response rates on the stimuli during acquisition over the last three sessions were similar as well (Figure 3C, target vs. CS1: *t*_(15)_ = 1.1; *p* = 0.281; target vs. CS2: *t*_(15)_ = 0.49; *p* = 0.629; CS1 vs. CS2: *t*_(15)_ = 0.03; *p* = 0.748).

### Extinction

Fractional response counts to the target did not differ significantly under saline or APV conditions respectively during extinction training (RMANOVA: saline: *F*_(5,75)_ = 1.5, *p* = 0.202, APV: *F*_(5,75)_ = 0.97, *p* = 0.442; Figure 3B). However, a two-way RMANOVA revealed a block effect (*F*_(5,75)_ = 2.4, *p* = 0.049) but neither treatment (*F*_(1,15)_ = 0.7, *p* = 0.41) nor interaction effects (*F*_(5,75)_ = 0.45, *p* = 0.82). Non-rewarded CS presentations led to decreased response probability under both saline (RMANOVA: *F*_(8,75)_ = 22, *p* < 10^−14^) and drug conditions (RMANOVA: *F*_(5,75)_ = 4.1, *p* = 0.002). A two-way RMANOVA revealed a treatment (*F*_(1,15)_ = 12.92, *p* = 0.003), block (*F*_(5,75)_ = 17.65, *p* < 10^−10^) and interaction effect (*F*_(5,75)_ = 5.1, *p* < 10^−4^). Paired *t*-tests showed significant differences in blocks 4–6 between CS_sal_ and CS_APV_ (*t*_(15)_ = 2.76; *p* = 0.014; block 5: *t*_(15)_ = 4.5; *p* = 0.0004; block 6: *t*_(15)_ = 3.56; *p* = 0.004). Importantly, fractional response counts for the target differed between drug conditions in the last block of extinction (paired *t*-test: *t*_(15)_ = 2.24, *p* = 0.04), hinting at an unspecific effect of APV on conditioned responding. Therefore, we proceeded to investigate this possibility using absolute response counts.

Figure 3D depicts the mean absolute response rates to the target and the CSs under saline and drug conditions during extinction. A two-way RMANOVA for target responses between the two conditions revealed no treatment (*F*_(1,15)_ = 1.9, *p* = 0.188) but a block effect (*F*_(5,75)_ = 5.7, *p* < 10^−3^), as well as a significant interaction of treatment and block factors (*F*_(5,75)_ = 6.8, *p* < 10^−4^). Follow-up RMANOVAs indicated that target responses increased significantly under APV (*F*_(5,75)_ = 10, *p* < 10^−6^) but not under saline (RMANOVA: *F*_(5,75)_ = 1.7, *p* = 0.143). Regarding responding to the CSs, a two-way RMANOVA yielded both significant treatment (*F*_(1,15)_ = 13.1, *p* = 0.003) and significant block effects (*F*_(5,75)_ = 14.6, *p* < 10^−9^), accompanied by a significant interaction (*F*_(5,75)_ = 2.8, *p* = 0.021). Follow-up RMANOVAs revealed significant response decrements to the CS in both conditions (CS_APV_: *F*_(5,75)_ = 3.5, *p* = 0.007; CS_sal_: *F*_(5,75)_ = 16, *p* < 10^−10^).

These results from fractional and absolute responses suggest that blocking NMDA-receptors of the NCL delays extinction learning. However, APV injection also increased responding to the (non-extinguished) target, indicating that the drug effect was not specific to the CS. To disentangle the non-specific response disinhibition from a potential addition effect on extinction learning, we conducted a series of pairwise comparisons to identify the time point at which a drug effect on target and CS responses could be demonstrated. Indeed, a paired *t*-test showed that absolute responding to the CS already differed between saline and drug conditions in block 4 (trials 13–16, *t*_(15)_ = 2.83, *p* = 0.03), while at that time responses to the target did not differ significantly between conditions (*t*_(15)_ = 1.86, *p* = 0.083). The lack of statistical significance was not due to a ceiling effect, as target responding for APV still increased significantly beyond this point (block 4 vs. block 6: *t*_(15)_ = 3.4, *p* = 0.004).

In another attempt to disentangle these two effects (slowed extinction and disinhibition), we calculated normalized response rates to the CS (Figure 3E). Normalization was performed by multiplying CS response counts by the ratio of target responses under saline to target responses under APV (see methods), with the intention to statistically remove the unspecific effect of APV on conditioned responding, as measured by the target control stimulus. Importantly, even when the non-specific increase in responding as measured by increased target responses was factored out through normalization of CS responses, differences between APV and saline remained: while the time course of the response decrement is highly similar between conditions, responding under APV is stronger than under saline, as indicated by a significant treatment effect (two-way RMANOVA: *F*_(1,15)_ = 10, *p* = 0.006; block: *F*_(5,75)_ = 14.3, *p* < 10^−9^; interaction: *F*_(5,75)_ = 0.3, *p* = 0.919). Similar to the previous analysis, responses to the CS under both conditioned started to differ after block 3 (paired *t*-test: block 4: *F*_(15)_ = 2.84; *p* = 0.012; block 5: *F*_(15)_ = 3.04; *p* = 0.008; block 6: *F*_(15)_ = 2.47; *p* = 0.03).

Taken together, the analyses of fractional response counts, absolute response counts, and normalized response counts support the hypothesis that APV, in addition to an unspecific enhancement of conditioned responding, specifically delays extinction learning.

### Retrieval

**Figure 4:**
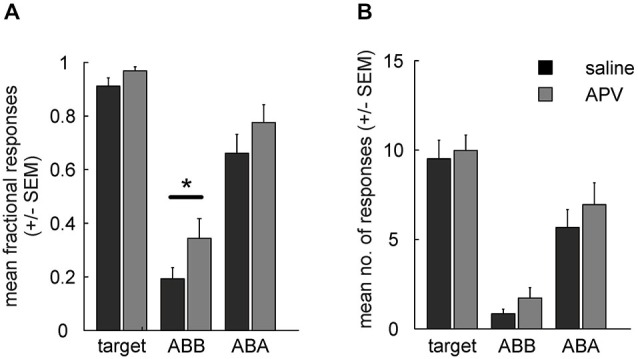
**(A)** Fractional response counts (±SEM) during retrieval testing. Significant difference in the ABB condition indicates impairment of extinction learning under APV. **(B)** As in **(A)**, but using mean absolute response counts. Asterisk indicates a significant difference (*p* < 0.05).

Retrieval of extinction memory was tested by presenting all stimuli in both contexts. Two-way ANOVA analysis for fractional CS responding in ABB and ABA revealed a main effects of (prior) treatment (*F*_(1,15)_ = 8.1, *p* = 0.01) and of testing context (ABB vs. ABA, *F*_(1,15)_ = 65.5, *p* < 10^−6^) in the absence of a significant interaction (*F*_(1,15)_ = 0.2, *p* = 0.68). *Post hoc* tests indicated that fractional CS response counts in the context of extinction differed significantly between drug conditions (ABB, extinction under drug vs. saline: *t*_(15)_ = 2.5, *p* = 0.025) while ABA renewal was unaffected (ABA: *t*_(15)_ = 1.7, *p* = 0.111) (Figure 4A).

For absolute response rates (Figure 4B) a two-way RMANOVA showed no significant main effect of treatment (*F*_(1,15)_ = 3, *p* = 0.105) or interaction (*F*_(1,15)_ = 0.1, *p* = 0.774), but there was a significant main effect of test context (block ABA vs. ABB: *F*_(1,15)_ = 37, *p* < 10^−4^). In contrast to fractional response counts, responding to the CS extinguished under saline was not significantly different from responding to the CS extinguished under APV when tested in the context of acquisition (ABA; *t*_(15)_ = 1.1, *p* = 0.297) or when tested in the context of extinction (ABB; *t*_(15)_ = 1.6, *p* = 0.132). Thus, fractional response counts again turned out to be more sensitive for detection of pharmacological manipulation than absolute response counts, as was found in our earlier study (Lengersdorf et al., 2014b).

Unimpaired renewal could in principle be due to a ceiling effect, i.e., that animals responded maximally during ABA testing under both drug and saline and therefore a possible effect on associative strength is masked. However, inspection of Figures 4A,B shows that ABA response counts to the CSs were somewhat lower than to the target, and statistical analyses indicated that the differences in response counts between CS and target were statistically significant in some cases and marginally significant in the others (fractional response counts: target vs. CS_APV_ in ABA: *t*_(15)_ = 2.06, *p* = 0.057; target vs. CS_sal_ in ABA: *t*_(15)_ = 4.39, *p* < 10^−4^; absolute response counts: target vs. CS_APV_ in ABA: *t*_(15)_ = 2.12, *p* = 0.051; target vs. CS_sal_ in ABA: *t*_(15)_ = 9.44, *p* < 10^−8^). We conclude that a ceiling effect is unlikely to have masked differential responding between APV and saline treatments in ABA testing.

## Discussion

The present study investigated the role of NMDARs in the NCL for extinction memory by pharmacologically modulating these receptors with the antagonist APV during extinction. In our previous study (Lengersdorf et al., 2014b) we reported that transient “prefrontal” NCL inactivation with the sodium channel blocker Tetrodotoxin (TTX) during extinction learning impairs extinction memory consolidation. Now, in APV-injected subjects, several analyses showed that extinction learning was slowed down through NMDAR antagonism injection. This effect was accompanied by general behavioral disinhibition, as evidenced by subjects’ enhanced responding to the continuously reinforced target stimulus. Context-dependent extinction memory retrieval revealed that the APV-treated animals did not exhibit a retrieval deficit as such but merely continued responding at the level of the last trials of extinction training.

Regarding the effects of APV, the present findings mostly align well with previous work from our laboratory. Lissek et al. (2002) demonstrated that NMDAR blockade in the NCL slows down color reversal learning due to prolongation of extinction. Our study likewise mostly accords with Lissek and Güntürkün (2003) who demonstrated that APV in the NCL retards extinction learning. However, Lissek and Güntürkün (2003) could not see a concomitant behavioral disinhibition of responding to a non-rewarded stimulus. This stimulus corresponds to our non-target and our results for this stimulus are identical to what was described by these authors (Lissek and Güntürkün, 2003). However, we additionally included a stimulus which was always followed by reward (target) and therefore consistently produced conditioned responding. Importantly, responding to this stimulus did increase under APV (during the last third of extinction training), suggesting that some of the effects of APV on responding to the extinguished CS are indeed due to behavioral disinhibition. However, fractional and normalized CS response counts indicated that disinhibition does not explain the full extent of the retardation of extinction. This pattern of results highlights the necessity to include appropriate control stimuli when applying pharmacological agents to animals, as unspecific effects on responding might otherwise be mistakenly attributed to specific learning mechanisms. Importantly, the presence of a significant difference between ABB CS response counts during retrieval reinforces our conclusion that APV does not merely disinhibit conditioned responding, but affects the encoding or the consolidation of extinction memory as well, because retrieval testing was conducted after any drug effects had dissipated.

It might seem counterintuitive that blocking NMDARs results in an increase rather than a decrease of behavioral output, since NMDAR activation depolarizes neurons due to influx of cations. However, blockade of NMDARs in PFC indeed does not dampen neural excitability but rather enhances it. For example, systemic MK-801 injections in rats impair working memory and, at the same time, increase motor activity, and the magnitude of these effects correlates with firing rate potentiation and burst activity reduction in the PFC (Jackson et al., 2004). MK-801 seems to act through decreased inhibitory interneuron activity, thereby disinhibiting prefrontal pyramidal cells (Homayoun and Moghaddam, 2007). It is conceivable that a similar mechanism might be at work in the pigeon NCL since electrophysiological and morphological analyses of NCL neurons indicate the existence of fast spiking neurons which resemble GABAergic interneurons of the mammalian telencephalon (Kröner et al., 2002) and which project to principal neurons. The absence of disinhibition for the non-target (see also Lissek and Güntürkün, 2003) could be due to a floor effect or might be related to the much stronger appetitive associative strength of the target that was constantly rewarded. Taken together, locally blocking NMDARs during extinction learning in the limbic and “prefrontal” caudal nidopallium slows down extinction learning, and disinhibits responses to rewarded stimuli.

Finally, extinction memory retrieval was tested under conditions of spontaneous recovery and renewal. Blocking NMDARs in the caudal nidopallium during extinction did not affect renewal but significantly increased spontaneous recovery when using fractional rather than absolute response rates. Impaired spontaneous recovery is readily explained by the impairment of extinction learning under APV. The fact that fractional but not absolute response rates yielded significant effects (although the analysis using the latter measure pointed into the same direction) was already observed in our previous study using TTX inactivation of the NCL (Lengersdorf et al., 2014b). This is somewhat puzzling since absolute response counts reflect the subject’s valuation of a given CS in a graded manner (Honig, 1962; Starosta et al., 2013), while fractional response counts omit the valuation but detect more sensible if extinction memory can be retrieved in general. Fractional response counts in addition largely omit this information by reducing a continuum of responding to a dichotomous measure. This could be explained if absolute response counts were a very coarse measure of variation which would largely reflect non-specific factors and therefore merely represent noise, which would be reduced by dichotomizing responses into presence or absence of conditioned responding.

But why did we observe a result pattern with APV that deviates from the TTX-results that were obtained with the identical design by Lengersdorf et al. (2014b)? In this first study, we found that TTX-injections into NCL do not impair extinction learning but rather impair extinction memory retrieval (Lengersdorf et al., 2014b). This accords with similar experiments on the PFC in mammals which make it likely that extinction learning can proceed without prefrontal involvement in various downstream neural structures (Burgos-Robles et al., 2007; Milad and Quirk, 2012). However, the retrieval of extinction memory requires that the PFC had modified its synaptic contacts with neurons that had undergone extinction learning (Milad and Quirk, 2002; Vertes, 2004; Herry et al., 2008). Consequently, impaired NCL/PFC-functions during extinction learning perturb subsequent extinction memory retrieval from downstream structures (Sierra-Mercado et al., 2006; Lengersdorf et al., 2014b). Here, using APV, we observe impaired extinction learning but no impaired extinction memory retrieval. As outlined above, our APV-injections possibly increased excitability of caudal nidopallial principal neurons. The NCL is one of the largest hubs of the bird forebrain and is connected to a very large number of sensory-associative, limbic and motoric areas (Shanahan et al., 2013). Possibly, an APV-induced increase of excitation of nidopallial principal neurons interferes with extinction learning in this wide forebrain network, resulting in slowed down extinction. At the same time, an increased excitation of nidopallial principal neurons could easily explain the selective disinhibition of responses to a reward-associated stimulus as observed in our study.

A large number of rodent studies suggest that blocking NMDARs results in a retardation of extinction learning (Baker and Azorlosa, 1996; Santini et al., 2001; Lee et al., 2006; Hsu and Packard, 2008). These results match our findings for context-specific extinction learning. Additionally, we could show that blockade of NMDA receptors results in behavioral disinhibition on top of its effects on extinction learning, and that our paradigm allows disambiguating these two effects.

To conclude, our results support the notion that NMDARs in the pigeon’s limbic and “prefrontal” caudal nidopallium is implicated in extinction learning as well behavioral inhibition. The comparative approach underscores the shared functionality of the NCL and the prefrontal areas of mammals and shows that the neurochemical architecture of extinction learning shows some invariant properties in vertebrates that are separated by 300 million years of independent evolution.

## Conflict of Interest Statement

The authors declare that the research was conducted in the absence of any commercial or financial relationships that could be construed as a potential conflict of interest.
